# Encapsulation of Electron Beam Melting Produced Alloy 718 to Reduce Surface Connected Defects by Hot Isostatic Pressing

**DOI:** 10.3390/ma13051226

**Published:** 2020-03-09

**Authors:** Yunus Emre Zafer, Sneha Goel, Ashish Ganvir, Anton Jansson, Shrikant Joshi

**Affiliations:** 1 Department of Engineering Science, University West, 461 86 Trollhättan, Sweden; sneha.goel@hv.se (S.G.); shrikant.joshi@hv.se (S.J.); 2 Research & Technology, Department of Process Engineering, GKN Aerospace Engine Systems AB, 461 81 Trollhättan, Sweden; ashish.ganvir@gknaerospace.com; 3 School of Science and Engineering, Örebro University, 701 82 Örebro, Sweden; anton.jansson.mail1@gmail.com

**Keywords:** electron beam melting, additive manufacturing, Alloy 718, surface defects, encapsulation, coating, hot isostatic pressing

## Abstract

Defects in electron beam melting (EBM) manufactured Alloy 718 are inevitable to some extent, and are of concern as they can degrade mechanical properties of the material. Therefore, EBM-manufactured Alloy 718 is typically subjected to post-treatment to improve the properties of the as-built material. Although hot isostatic pressing (HIPing) is usually employed to close the defects, it is widely known that HIPing cannot close open-to-surface defects. Therefore, in this work, a hypothesis is formulated that if the surface of the EBM-manufactured specimen is suitably coated to encapsulate the EBM-manufactured specimen, then HIPing can be effective in healing such surface-connected defects. The EBM-manufactured Alloy 718 specimens were coated by high-velocity air fuel (HVAF) spraying using Alloy 718 powder prior to HIPing to evaluate the above approach. X-ray computed tomography (XCT) analysis of the defects in the same coated sample before and after HIPing showed that some of the defects connected to the EBM specimen surface were effectively encapsulated by the coating, as they were closed after HIPing. However, some of these surface-connected defects were retained. The reason for such remnant defects is attributed to the presence of interconnected pathways between the ambient and the original as-built surface of the EBM specimen, as the specimens were not coated on all sides. These pathways were also exaggerated by the high surface roughness of the EBM material and could have provided an additional path for argon infiltration, apart from the uncoated sides, thereby hindering complete densification of the specimen during HIPing.

## 1. Introduction

Additive manufacturing (AM), also commonly known as three-dimensional (3D) printing, is a rapidly growing technology for generating complex geometrical products layer by layer from a 3D computer-aided design (CAD) model data [1,2]. This technology offers significant design freedom compared to conventional manufacturing methods such as forging, casting, etc. [3]. Electron beam melting (EBM) is one of the AM techniques; it utilizes an electron beam as the heat source [4]. Manufacturing of difficult-to-machine nickel-based superalloys, such as Alloy 718, by using EBM is being explored and has attracted significant interest in the aerospace industry due to reduced raw material wastage, which leads to lower costs and buy-to-fly-ratio, and less contamination due to vacuum conditions during the process [5]. 

However, EBM-manufactured Alloy 718 is typically characterized by the presence of inevitable defects such as lack of fusion, gas porosity, and shrinkage porosity, which can be detrimental to the mechanical properties of the material [6]. Therefore, thermal post-treatment involving hot isostatic pressing (HIPing) is typically employed to reduce such defects in the AM manufactured material [3,7,8,9]. In this context, it is pertinent to state that micro-computed tomography is used to obtain detailed information about the location and size of defects present in the material in 3D [10,11,12]. It has been reported that HIPing can heal most defects in AM manufactured specimens, except the surface-connected defects [11,13]. The presence of these defects becomes vital, as they are not affected by HIPing and are found to be potential crack initiation sites leading to fracture of the material under fatigue loading [6]. Therefore, an idea of encapsulating the surface-connected “open” defects through deposition of a thin film/coating on the as-built specimen surface was explored in the literature for laser-based AM techniques [13]. However, this approach has not yet been widely investigated, and in particular no such efforts have yet been reported in case of EBM-manufactured material, where retained surface-connected defects after HIPing can be of concern. Therefore, the challenges presented in the above led to a hypothesis that is tested in the present work. The hypothesis is that encapsulation of these surface defects by applying a coating on the EBM-built Alloy 718 could allow HIPing to effectively heal all defects present, including those that are surface-connected.

In the present study, the coating technique utilized to explore the encapsulation hypothesis is high-velocity air fuel (HVAF), which is one of the thermal spray coating techniques [14,15]. Some of the EBM-manufactured Alloy 718 specimens were coated. Afterward, the coated and uncoated specimens were subjected to HIPing to enable a comparison of extent of defect closure in the two conditions. A detailed investigation of the defects in the specimens is carried out by using light optical microscopy (LOM), scanning electron microscopy (SEM), and X-ray computed tomography (XCT). In addition, surface roughness of the as-built specimen is measured using white light interferometry.

## 2. Materials and Methods 

### 2.1. EBM Processing

The feedstock material used for EBM production was plasma-atomized Alloy 718 powder supplied by Advanced Powders and Coating (Québec, Canada). The nominal powder particle size range was 45–105 µm, and its chemical composition, as provided by the supplier, is given in Table 1. The powder was recycled several times before usage.

**Figure 1:**
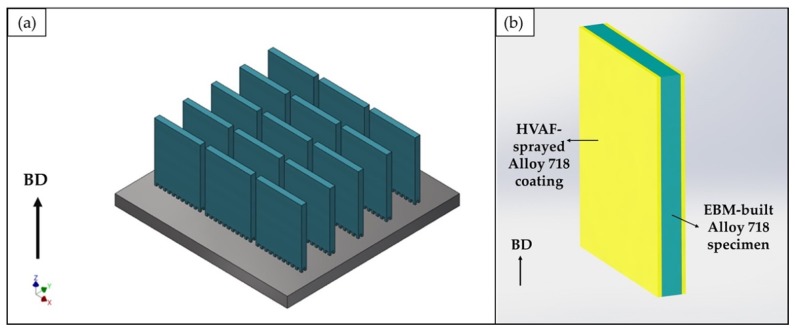
(**a**) Image of the computer-aided design (CAD) model of the entire electron beam melting (EBM) build, and (**b**) illustration of the coated specimen. The arrow indicates the build direction (BD).

An Arcam A2X EBM machine was used to produce several rectangular specimens of dimensions 53 × 45 × 5 mm, as shown in Figure 1a. Typical process parameter settings recommended by Arcam AB (Mölndal, Sweden) were used (EBM control software version 4.2.76). The build process started after preheating the stainless-steel build plate to ~1025 °C. A layer thickness of 75 µm and an acceleration voltage of 60 kV were used. The periphery of the specimens, also termed as contour, was melted using multispot melting. The interior of the specimens, known as hatch, was processed using back and forth raster scanning. During hatch melting, the scan direction was rotated by 72° for every added layer.

### 2.2. Encapsulation Concept

The feedstock material used for coating was Alloy 718 powder manufactured by Praxair Surface Technologies (Indianapolis, USA). The powder particle size range was 15–45 µm, and its chemical composition, as provided by the supplier, is listed in Table 2. Half of the as-built EBM Alloy 718 specimens were coated on the back and front sides (53 × 45 mm faces), leaving the remaining sides uncoated, as shown in Figure 1b. The coating was deposited using an HVAF M3 system (Uniquecoat, Richmond, USA) with a target coating thickness of about 500 µm. Although substrates to be HVAF sprayed are typically grit blasted to a roughness of about 5–10 µm arithmetic mean roughness (Ra), it is important to point out that there was no surface preparation done prior to coating deposition on the EBM specimens, which already had an average surface roughness of about 80 µm arithmetic mean height (Sa) in as-built condition.

### 2.3. Hot Isostatic Pressing

Some of the specimens in as-built and coated conditions were HIPed in a QIH21 model molybdenum HIP furnace at Quintus Technologies (Västerås, Sweden). HIPing was carried out at a temperature of 1,120 °C, and pressure of 100 MPa was applied for 4 h. Argon was used as an inert process gas. After the dwell time, the specimens were rapidly cooled.

### 2.4. Metallographic Preparation and Characterization

For microstructural investigation, samples were sectioned along and perpendicular to the build direction with an alumina abrasive cutting disc mounted on a Struers Secotom 10 machine. The sectioned samples were hot mounted using a Buehler SimpliMet 3000 press. The mounted samples were grinded using silicon carbide paper ranging from P360 to P1200 grits, followed by polishing with 9 µm and 3 µm diamond suspensions, and thereafter with 0.05 µm colloidal silica suspension. Grinding and polishing were done using a semi-automatic Buehler Ecomet 300 Pro machine. 

**Figure 2:**
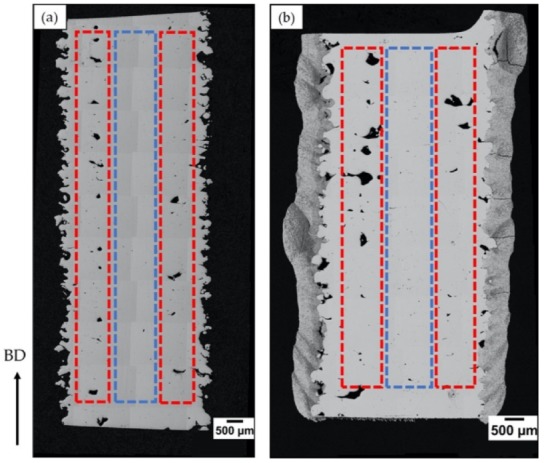
Light optical microscopy (LOM) micrographs of the specimen cross-sections in the (**a**) uncoated and (**b**) coated conditions. The dotted red and blue lines indicate the regions of contour and hatch, respectively, distinctly analyzed for defect quantification. The arrow indicates the build direction.

The defects, particularly their type, amount, and distribution, were analyzed at suitable magnifications using Zeiss Axio light optical microscope. The defect content in the hatch and contour regions were distinctly evaluated using the LOM micrographs, which were processed by using image analysis software ImageJ. In each case, no less than 15 LOM images captured along the cross-section (see Figure 2) were analyzed to obtain representative defect content. Hitachi TM3000 scanning electron microscope was employed to perform a high magnification analysis of the coating. The porosity of the coating, before and after HIPing, was evaluated by image analysis software ImageJ, using no less than 15 SEM micrographs captured along the cross-section to get a representative value of the porosity content.

For a further precise analysis of the defects, XCT was employed. For this, a sample of dimensions 10 × 10 × 6 mm was sectioned out. The defects present in the specimen, including their size, shape, and location, were characterized using a Zeiss Xradia Versa 520 XCT system. XCT scans were performed over the entire volume of the same coated specimen (10 × 10 × 6 mm) before and after HIPing.

The surface roughness of the as-built specimen was characterized through white light interferometry using a profilm3D (Filmetrics, San Diego, CA, USA). The surface roughness parameter, Sa, was evaluated from the 3D topography maps of the specimen. For each measurement point, an area of 1 mm by 1 mm was analyzed. In total, six such measurements were performed over the as-built specimen.

## 3. Results and Discussion

### 3.1. Uncoated Condition

**Figure 3:**
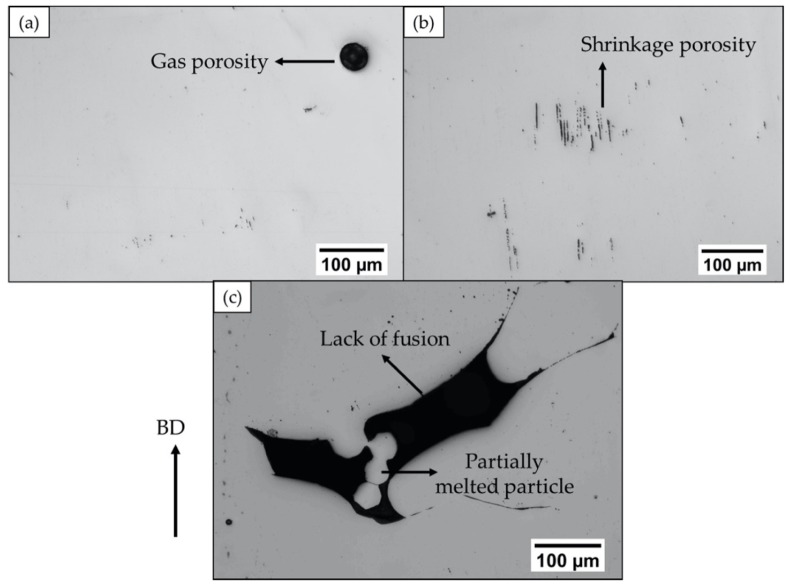
LOM micrographs showing the different kinds of defects present in the as-built EBM Alloy 718: (**a**) gas porosity, (**b**) shrinkage porosity, and (**c**) lack of fusion. The arrow on the left indicates the build direction.

The as-built EBM Alloy 718 specimens were characterized by the presence of defects. Three types of defects were observed, i.e., lack of fusion, shrinkage porosity, and gas porosity, and they are visualized in Figure 3. Such defects have also been previously observed by Goel et al. [3], and the influence of the defects on mechanical properties has been elaborated elsewhere [16,17]. Round-shaped gas porosities (refer Figure 3a) were randomly distributed, and they are attributable to entrapped gas inside the virgin powder, which could have found its way into the EBM build [1]. The shrinkage porosities, shown in Figure 3b, were typically aligned along the build direction, and are reportedly known to form as a result of interdendritic shrinkage after solidification [18]. The lack of fusion defects were primarily concentrated in the contour regions and often contained partially molten powder particles. The main reason for the formation of lack of fusion defects is expected to be inappropriate energy input. Low energy input can result in incomplete bonding between the layers [19], and, as a result, partially molten particles can be observed in these defects (see Figure 3c). On the other hand, high energy input can cause the melt to spatter away [19].

**Figure 4:**
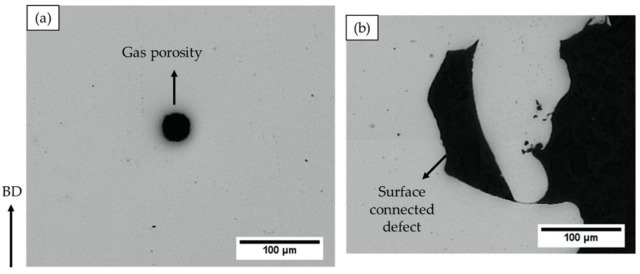
LOM micrographs revealing the remnant defects in the hot isostatic pressed (HIPed) EBM Alloy 718: (**a**) gas porosity and (**b**) lack of fusion. The arrow on the left indicates the build direction.

The as-built specimen was also subjected to HIPing, which caused a significant reduction in defect content. A closer investigation of the entire HIPed specimen revealed that nearly all the shrinkage porosities were healed. However, some of the gas porosities and lack of fusion defects were present after HIPing, as shown in Figure 4. A possible explanation for remnant gas porosities after HIPing could be entrapped argon gas, which exerts opposite pressure and can hinder complete closure [6]. However, the defects of typically more serious concern are lack of fusions [6]. A vast majority of remnant lack of fusion defects were found to be surface-connected (open), as exemplified in Figure 4b; therefore, these were not healed after HIPing. This is consistent with several other studies by Tammas-Williams et al. [11] and Tillmann et al. [13], in which XCT was utilized to investigate the effects of HIPing on the defects in EBM-built materials, and it was observed that the remnant lack of fusion defects open to the surface were not closed after HIPing. The reason for this is pressure equalization inside and outside the defect, which can prevent it from closing [13]. In the case of EBM-built Alloy 718, Kotzem et al. [20] have also observed the presence of surface-connected defects. Therefore, to close such defects in EBM Alloy 718, the specimens were coated and HIPed.

### 3.2. Coated Condition

**Figure 5:**
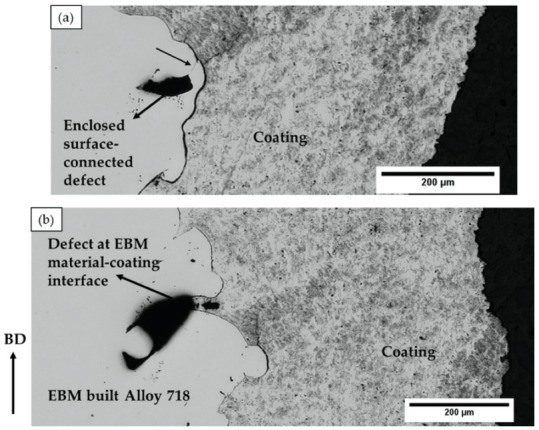
LOM micrographs showing (**a**) an enclosed defect in the EBM material and (**b**) a defect at the EBM material-coating interface in the as-built condition. The arrow on the left indicates the build direction.

**Figure 6:**
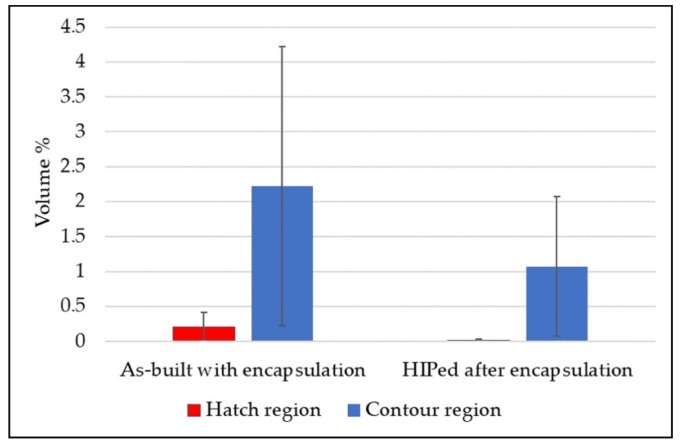
Defect content in the coated condition measured in the hatch and contour regions along the build direction.

The as-built EBM Alloy 718 specimens (~53 mm × 45 mm × 5 mm) were coated in an effort to enclose the surface-connected defects, as shown in Figure 5a. Some of the coated specimens were then subjected to HIPing to heal the defects present in EBM Alloy 718. The volume fraction of defects in hatch and contour regions in the coated condition before and after HIPing, as evaluated by image analysis, is given in Figure 6. It can be seen that the defect content was significantly reduced in both the hatch and contour regions after HIPing. However, full densification after HIPing could not be achieved as hypothesized at the beginning of this study. Some of the lack of fusion defects, specifically those in the contour and close to the surface, and gas porosities were found to persist even after HIPing the coated specimen. A post mortem study revealed that the coating seemed unable to completely seal the EBM specimen surface, as several defects at the EBM material-coating interface were observed (see Figure 5b).

**Figure 7:**
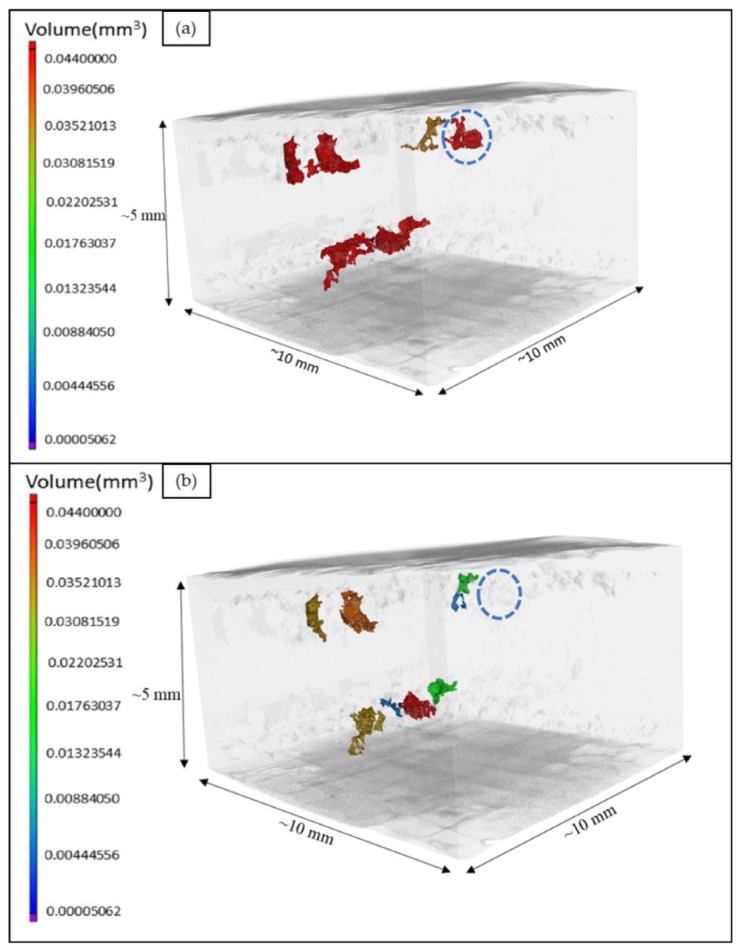
Three-dimensional (3D) views of large defects in the same coated sample (**a**) before and (**b**) after hot isostatic pressing (HIPing), obtained from x-ray computed tomography (XCT) analysis.

In order to get a more detailed understanding of the above observation, especially to identify surface-connected defects, further XCT analysis was carried out. A coated sample was analyzed before and after HIPing to precisely track the defects in the two conditions through XCT. The larger lack of fusion defects, which had a connection to the EBM specimen-coating interface, were separately analyzed, as shown in Figure 7. The defect close to the EBM specimen surface marked with the blue dotted circle in Figure 7a appeared to be fully closed after HIPing, or at least reduced to a size below the resolution limit of XCT. This is attributable to the successfully complete encapsulation of this otherwise surface-connected defect, which could be healed during HIPing. However, some other defects appeared to be not fully closed. This could be possibly attributed to incomplete encapsulation of the defect, as discussed later. The defects were further individually analyzed using 2D slices from the XCT data.

**Figure 8:**
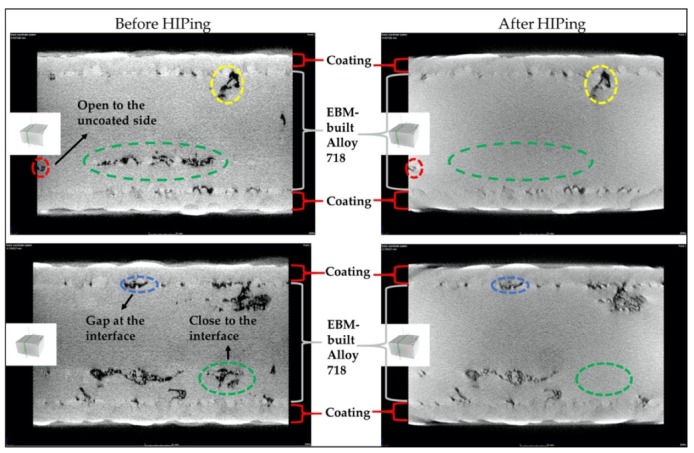
Two-dimensional (2D) sliced images from XCT analysis showing the “tracked” defects at the same cross-sections in the entire coated sample before and after HIPing.

Figure 8 shows a comparison of defects present at identical cross-sections of the same coated EBM specimen before and after HIPing as obtained from the XCT analysis. It is important to mention that specimens were only coated on the front and back sides as shown in Figure 1b. Some defects, for instance, the one marked with red dotted lines in Figure 8, can be clearly seen to be open to the uncoated side. It was observed that most of the lack of fusion defects that appeared to have a connection to the EBM specimen-coating interface did not close after HIPing (highlighted with yellow dotted lines in the figure). Nevertheless, some of the lack of fusion defects that were close to the interface seemed to be healed (marked with green dotted lines). Moreover, most of the gaps at the interface of EBM-built material and coating remained unhealed (marked with blue dotted lines in Figure 8). This can be explained.

**Figure 9:**
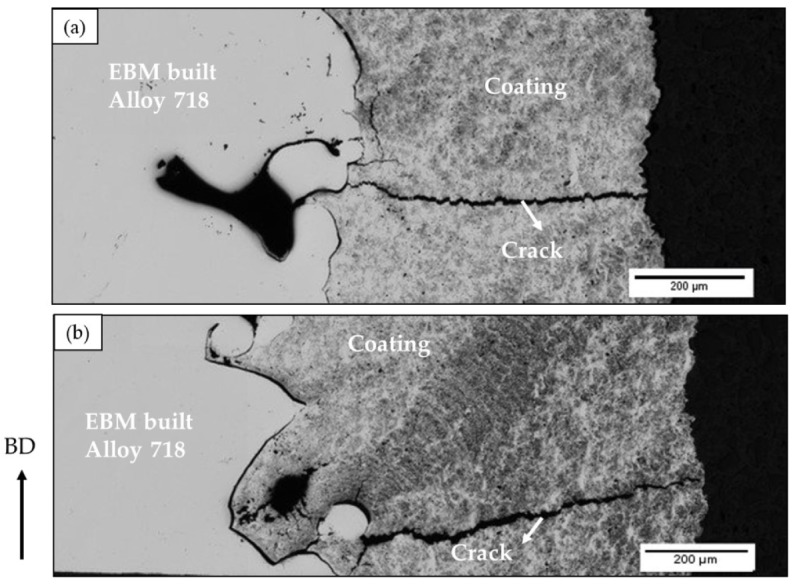
LOM micrographs showing (**a**) a crack in the as-built sample and (**b**) a remnant crack present in the HIPed sample.

**Figure 10:**
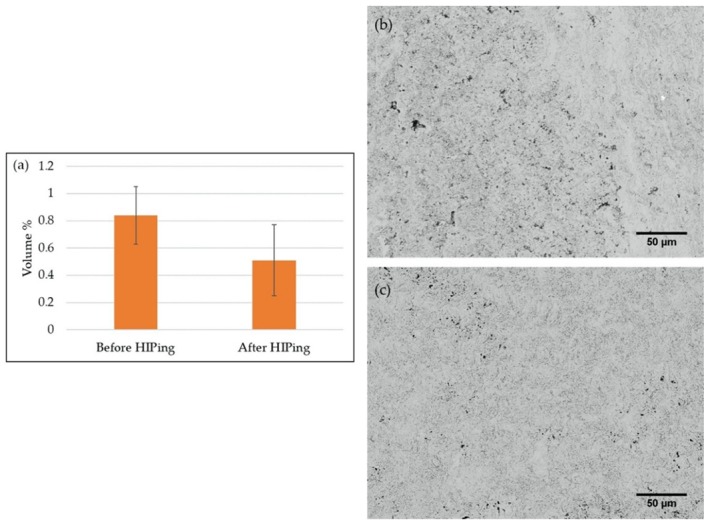
(**a**) Porosity content in the coatings before and after HIPing, as determined by image analysis, SEM micrographs of the coating (**b**) before HIPing, and (**c**) after HIPing.

The interface between EBM specimen and coating exhibited gaps, as shown in Figure 5b, which could be attributed to the high surface roughness of the as-built EBM Alloy 718. It is pertinent to note that the coating was applied on the as-built surface without any prior surface preparation. The surface roughness value of the as-built EBM specimen was nearly 80 µm Sa, as measured by white light interferometry, and such high roughness could have hindered complete sealing of the specimen surface. In this context, it is worth mentioning that, although it is necessary to have an appropriately rough substrate to be used for any thermal spray coating process, to achieve better coating-substrate adhesion, an excessively high surface roughness can introduce defects at the substrate-coating interface by not permitting the molten splats to completely fill the “valleys” in the existing surface asperities (see Figure 5b). In the present study, the coating was deposited on a rather rough surface (Sa ~ 80 µm) compared to what is typically reported in the literature, i.e., about 10 µm [21,22,23,24]. In addition to these interface defects, several through-thickness vertical cracks were observed in both the coated samples, i.e., HIPed and not HIPed condition, as shown in Figure 9, which could also be possibly attributed to the high surface roughness. The total porosity content of the coatings before and after HIPing, as evaluated by image analysis, is shown in Figure 10. It can be seen from the figure that HIPing resulted in porosity reduction by an order of magnitude. However, some amount of porosity was present after HIPing. The vertical cracks, gaps at the EBM specimen-coating interface, and the porosity in the coating could have provided the path for argon gas infiltration inside the EBM specimen during HIPing. This could possibly explain why some of the defects, mainly lack of fusions connected to the EBM specimen-coating interface, and the gaps at the interface were still present in the coated and HIPed EBM specimen.

## 4. Summary and Conclusions

In this study, the efficacy of encapsulating the as-built EBM Alloy 718 specimens was investigated to eventually close surface-connected defects after HIPing. The major findings of the study based on the obtained results are as follows:

- The hypothesis that encapsulation of EBM specimens through coatings can eliminate surface-connected defects during subsequent HIPing presents a novel idea. However, it could only be partly tested in this paper due to (a) very large surface roughness of the as-built EBM 718 specimen used for this study and (b) only two sides of the as-built specimen being coated.
- Few of the surface-connected defects were closed after subjecting the coated EBM-built specimen to HIPing. However, some of the lack of fusion defects and gaps at the EBM specimen-coating interface remained after HIPing.
- The presence of defects in the coated and HIPed specimen was rationalized as follows: the high surface roughness of the as-built specimens caused only partial “sealing” of defects, as gaps were observed between the EBM specimen and the coating. In addition, the through-thickness cracks resulting during coating on very rough substrate surfaces could have also connected the defects to the surface, despite the application of coating.
- The specimens were coated on only the two larger faces, leaving the remaining sides uncoated. This could have provided an additional path for HIP process gas infiltration from the uncoated sides.

It is inferred that the surface roughness of the EBM specimen, prior to coating deposition, should be reduced to enable complete sealing of the surface-connected defects. Thus, before coating, prior surface preparation by mechanical post-treatment techniques, such as shot peening, machining, and grit blasting, can be used in cases where the as-built surface roughness is very large. Encapsulation of EBM-built materials should be done on all the sides of the specimens, as it can enable more effective defect closure during HIPing.

## Figures and Tables

**Table 1:** Nominal chemical composition of Alloy 718 powder used during electron beam melting (EBM).

Element	Ni	C	Cr	Mo	Ti	Al	Fe	Nb	Ta
Wt %	54.10	0.03	19.00	2.90	1.00	0.50	Bal.	4.90	<0.01

**Table 2:** Nominal chemical composition of Alloy 718 powder used for coating.

Element	Ni	C	Cr	Mo	Ti	Al	Fe	Nb + Ta
Wt %	50.00–55.00	0.02–0.08	17.00–21.00	2.80–3.30	0.70–1.10	0.03–0.70	15.00–21.00	4.70–5.50

## Data Availability

The raw data required to reproduce these findings cannot be shared at this time, since the data also form part of an ongoing study in the author’s research group.
